# The Surgery of Joints

**Published:** 1891-06-06

**Authors:** 


### The Practitioner's Mirror and Retrospect.

[*The Editor will be glad to receive offers of co-operation and contributions from members of the profession. All letters should be addressed to* The Editor, The Lodge, Porchester Square, London, W.]

### SURGERY.

### THE SURGERY OF JOINTS.

The doctrines of antiseptic and aseptic surgery opened a new era in the treatment of injuries and diseases of joints. Compound fractures or dislocations of joints are now treated in the same way as similar injuries affecting the continuity of a bone. By the aseptic method surgeons now open a joint with safety to remove loose bodies, or to fix a movable semilunar cartilage in the knee, or simply to drain an obstinate hydrops. Or again, the joint is incised to tap an abscess, to remove a slice of pulpy membrance, or, finally, to allow of the various procedures classed under the head of arthrectomy or erasion of a joint.

All these outshoots of the aseptic system are more or less fully recognised by the majority of the profession, but a most interesting and valuable paper by Mr. Watson Cheyne in the *British Medical Journal*, of March 7th, comes to remind us that there are still other logical and legitimate channels in which its beneficial influence may extend.

We will first call attention to a sentence in the opening part of the paper just referred to: "Nor have I," says Mr. Cheyne, "included operations for recent fracture of the patella or olecranon. . . . for operative interference in these cases is looked on now by a number of surgeons as the proper immediate treatment; it is the treatment which I always adopt, and I have seen no reason to abandon it."

Here then is a channel which might be more widely utilised. With some surgeons, as Mr. Cheyne says, the immediate wiring of a fractured olecranon or patella has come to be as much an axiom of practice as the opening of an abscess, and these, we doubt not, see no reason to abandon their custom; from others, however, this treatment has received but a cold reception. What is the reason of it? Is it want of faith in the system which has made such treatment feasible? Then all primary major operations which are not absolutely necessary should be equally excluded from the practice of such. Or is it that they claim to get equally good results by simpler means? If this be so, it were well that some clear demonstration of such results should be forthcoming. For our part we have seen no results from the simple methods of treatment that have stood the test of time —except *one*. This was a man with an apparently normal patella, which he said had been broken some thirty years previously. The movement and utility of the limb was perfect—but it is to be noted that the condition of the fracture at the time is unknown. There are undoubtedly cases—and we have seen such—where the evidence of fractured patella or olecranon is conclusive, and yet there is scarcely any separation of the fragments—which is due to a part of their aponeurotic covering being untorn.

Such cases, of course, do not belong to the class we are discussing, where there is an interval between the fragments which cannot be maintained in close enough apposition to allow of bony union, and hence a merely fibrous repair takes place, which is gradually stretched by the movements of the limb, considerably weakening it. Here, by wiring together the two fragments, apposition is complete, and true bony repair is obtained; an operation which is quite safe when carried out with due precautions, and leaves a strong and freely moveable joint—as the experience of Lister, Cheyne, and many others amply testifies. The wire often has to be removed after union has taken place, because its twist irritates the skin, but this is quite immaterial.

Having thus cleared the way, we may now follow Mr. Cheyne in the subject of his paper—the treatment by immediate operation of comminuted or obscure fractures into a joint, locking its movements, which cannot be restored by manipulation. The future condition of the limb in such cases is, as a rule, most unsatisfactory, the movement being very limited, and often in the case of the elbow joint requiring a subsequent excision to make the arm serviceable.

In such cases it is proposed to open the joint, remove any small detached fragments that are only in the way, clear away clots, and finally wire or peg together any portions of bone that can be usefully brought together.

This proceeding will remove the locking of the joint, replace moveable fragments, such as the olecranon, clear away clots, and prevent widespread adhesions taking place.

The cases related—partial fracture of head of radius, fracture of neck of femur, fracture of olecranon and coronoid processes and external condyle of humerus, with dislocation backwards, and fracture of the edge of an old standing dislocation of the patella—are a sufficient indication of the range of the treatment proposed.

The one requisite for its successful practice, besides ordinary surgical skill, is strict asepticism.

To all who have seen cases of fixed and distorted joints from old injury, the new method of treatment here advocated will come as a welcome addition to our means of usefulness. Its application is limited by its demanding access to full antiseptic appliances, and the security which habitual use of such appliances gives, but only so far.

